# IJN is now PubMed indexed

**DOI:** 10.4103/0971-4065.62085

**Published:** 2010

**Authors:** Vinay Sakhuja

This issue marks the start of the twentieth year of publication of the Indian Journal of Nephrology (IJN). Since its inception, members of our society have often wondered whether it would be possible to get the journal indexed with the United States National Library of Medicine so that the journal would be available on PubMed, the best known biomedical database. My team and I have been working towards this goal since January 2006, when I took over as Editor of the Journal.

We made several changes, which included entrusting the task to professional publishers: Medknow Publications. Getting a professional organization on board brought several advantages that included accurate verification of references and having the services of experienced copy editors. Another major change was to bring the process of manuscript submission and review in the digital age by making the whole process web-based, through the portal http://www.journalonweb.com/ijn. The process of submission, evaluation as well as production is practically paperless. Not only has this change been environment-friendly, but it has also been appreciated by most contributors. This almost immediately broadened the geographic area from where contributions came to the journal. The journal now regularly publishes articles from all continents. A locator map indicates the location of the author institute in each issue. The increase in number of contributions allowed us to focus more on quality. An indicator is the acceptance rate, which was only 40% in 2009.

The journal website (http://www.indianjnephrol.org) also underwent significant changes. It now provides both hypertext markup language (html) and portable document format (pdf) versions of all papers. You can now receive a table-of-content alert when a new issue comes online. The issues are now published well in advance of the print publication. Society members and subscribers can suggest changes to their mailing addresses from the journal's website.

It gives me great pleasure to inform all members that the national Library of Medicine, USA, has agreed to include the Indian Journal of Nephrology in PubMed and PubMed Central. One can now search on PubMed for the full text of all articles published in the Journal from 2008 onwards. These articles are also linked from PubMed to the journal's website. Older issues would also be made available from PubMed.

This is an important milestone for all of us since this broadens the visibility of all published articles. Also, we will now be able to attract original articles of even better quality, which were earlier being sent elsewhere. We hope to continue to improve the quality of papers by ensuring rigorous peer-review and on-time publication. In case a sufficient number of accepted manuscripts are available we may, in the near future, increase the number of articles published in each issue and thus shorten the time gap between acceptance and publication.

Your suggestions for further improvement of IJN are always welcome.

